# Curriculum delivery in Medical Education during an emergency: A guide based on the responses to the COVID-19 pandemic

**DOI:** 10.15694/mep.2020.000069.1

**Published:** 2020-04-16

**Authors:** Mohamed H. Taha, Mohamed Elhassan Abdalla, Majed Wadi, Husameldin Khalafalla

**Affiliations:** 1 College of Medicine and Medical Education Centre; 2 College of Medicine; 3 College of Medicine

**Keywords:** social distancing, COVID-19, online learning, distant learning

## Abstract

This article was migrated. The article was marked as recommended.

The spread of coronavirus (COVID-19) has led the majority of countries worldwide to implement emergency lockdown plans to limit the spread of the virus; this has resulted in the interruption of on-campus school and university instruction. Responses to the COVID-19 pandemic in medical education have varied from country to country, from closures of medical schools to online/distance learning approaches to abiding by country-specific measures such as social distancing to stop the spread of the disease.

The sudden transition from on-campus learning to exclusively distance learning is challenging for both faculty and students and has required a lot of preparation and other efforts in a short time. This paper aims to share the experiences of four authors in the middle east that have dealt with the sudden transition from ordinary teaching and learning to fully online teaching.

The process of Curriculum delivery in Medical Education during an emergency has included; establishing a sense of urgency, establishing working teams, conducting needs assessments, developing implementation plans, communicating the curriculum content, capacity building, managing students’ stress, finding tools to be used, managing student engagement and motivation, student assessment, anticipating challenges and planning for how to overcome them, and monitoring and evaluation of curriculum implementation and continuous improvement.

The proposed process will hopefully assist the medical schools in response to the current pandemic (COVID-19) and when facing similar situations.

## Background

Coronavirus, or COVID-19, is classified as a pandemic affecting 199 countries and territories around the world ( *COVID-19 Coronavirus Pandemic*, 2020; Sun *et al.*, 2020). The spread of COVID-19 has led a majority of countries worldwide to implement emergency lockdown plans and apply social distancing strategies to limit the virus’ spread; this has resulted in the interruption of school and university attendance ( Hafiz *et al.*, 2020). It has been estimated that there are 1,5 billion learners affected by school and university closures ( UNESCO, 2020). The responses to the COVID-19 pandemic/threat by medical schools around the world have varied, from total study cessation to a switch to online/distance learning. This sudden transition to distance learning approaches from on-campus learning is challenging for both faculty and students and has required much planning over a short period of time and without any clear guidelines.

Medical schools’ responses to these sudden lockdowns caused by COVID-19 are not well documented in the literature, and there are few works that outline the response to epidemics like these, such as he Severe Acute Respiratory Syndrome (SARS) outbreak. In 2000, some Chinese medical schools responded to SARS by shifting to online problem-based tutorial sessions (Savin-Baden, 2007), while in 2003, Chinese medical schools cancelled formal bedside teaching and postponed their exams ( Patil and Chan Ho Yan, 2003). The Canadian response to SARS included the suspension of clinical clerkships and electives for students for up to six weeks ( Clark, 2003).

**Figure 1.**
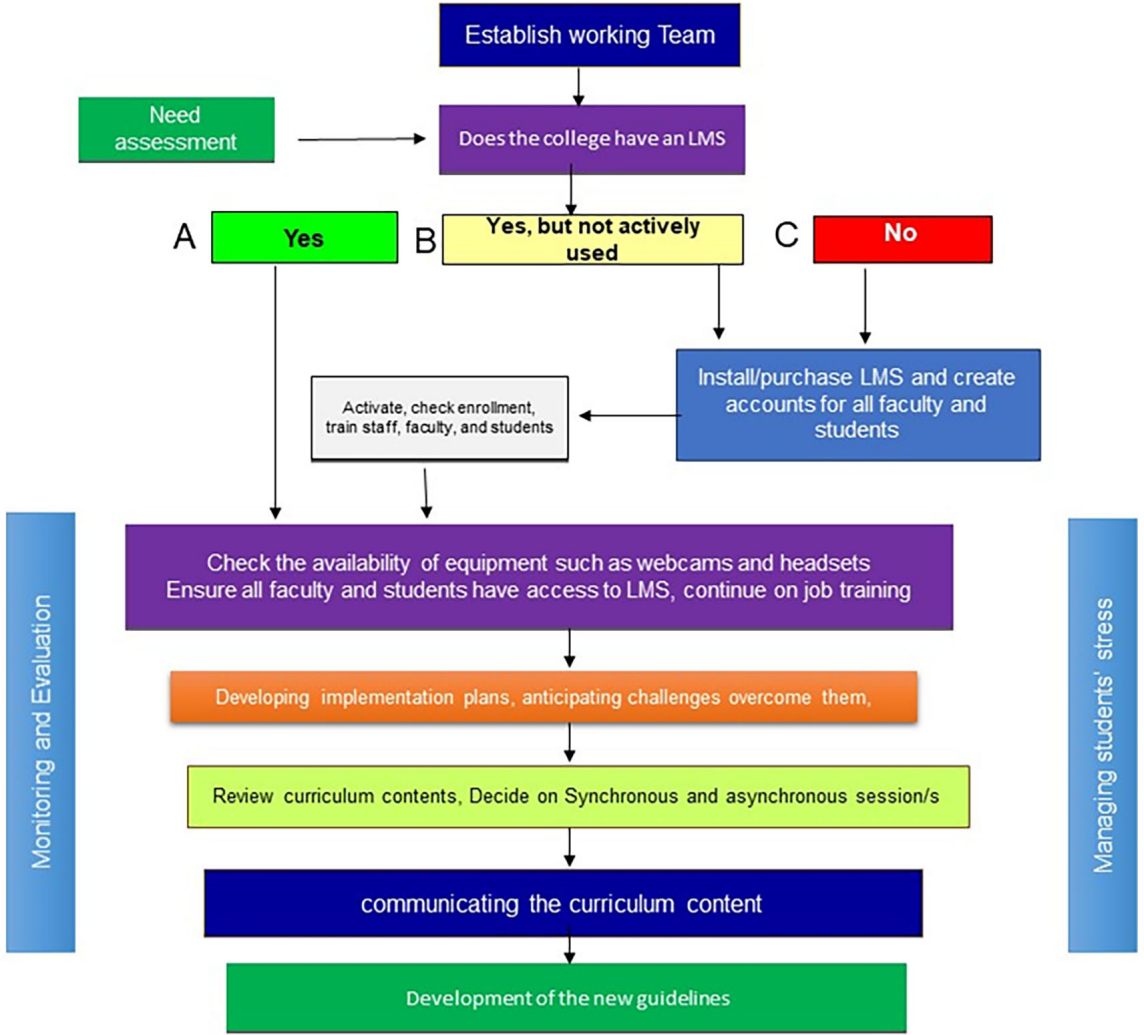
Guidelines for the implementation plans for three scenarios (A) if the college has an LMS; (B) if the college has a non-functional LMS; and (C) if the college has no LMS

In this paper, the authors provide guidelines that may facilitate dealing with the sudden transition from on-campus teaching and learning to online teaching in the light of both COVID-19 and similar situations. These guidelines stem from the real experience and reflection of the authors. Hopefully to be useful for the medical schools worldwide in the period of COVID-19 and the other similar situations. The guidelines will be presented under different headings and summarized in Figure 1 below:

## Establishing a sense of urgency

The first step to dealing with the shift from traditional curriculum delivery to online teaching is to maintain a semi-equal sense of urgency among faculty members and those who are involved in curriculum delivery. Leaders can do this by referencing both the effect of the pandemic on health and the specific effect of lockdown strategies on medical students (for example, reduced clinical exposure in specific specialities, causing a detrimental effect on competency attainment and future exam performance for students and junior doctors) ( Ahmed, Allaf and Elghazaly, 2020).

A comprehensive discussion of possible alternative means of communication (since face-to-face meetings may not be possible) for all faculty and administrators must be held in order to introduce the need for continuing the learning process using an online/distance learning approach. Building this sense of urgency is essential to change management ( Pollack and Pollack, 2015). It is crucial that this step is led by senior educational leaders at the school.

## Establishment of a working team

The function of a working team is to lead the planning, implementation, monitoring, and evaluation of the transition to online/distance learning. The team should include a team leader - preferably the head of the curriculum committee - and medical education experts, as well as course directors, learning management system officers (if applicable) or information technology officers, and student representatives, if possible.

The team should work with a suitable level of flexibility, clear terms of reference that relate to the task of managing the transition to distance learning, and clear lines of reporting and communication.

## Conducting a needs assessment

In situations where social distancing is required, there are three scenarios for implementing distance learning approaches (See Figure 1 above). The first is for colleges that already have a Learning Management System (LMS) or other learning application that is frequently used by all faculty and students. The second scenario is for colleges that have a Learning LMS but with a minimal use or non-functioning LMS.

The third scenario is for colleges that uses no elements of distance or online education.

A needs assessment should be conducted that includes the following and other assessment topics: the knowledge and skills of faculty members in modes of online/distance learning; the available resources and equipment, such as PCs, webcams, headsets, etc.; connectivity and access to the LMS for all students and faculty; and perhaps the rules and regulations that are in place, e.g. assessment methods or completion requirements.

## Setting the plan

The working team should then develop an implementing plan that considers the type of the curriculum, the academic level of courses, and the results of the needs assessment. The plan should start with faculty development and the curriculum changes The implementation should include timetables, as well as delivery methods for e-lectures, e-practical labs, e-problem-based learning (e-PBL), clinical skills, and clinical teachings.

The team should settle on which LMS or application will be used. There are several options for LMS used in medical education worldwide, with the most popular being Moodle and Blackboard. The latter has shown its effectiveness over time as the primary LMS used by medical schools ( Baig, Gazzaz and Farooq, 2020). It is, however, also important to think about alternatives such as Microsoft Teams and Zoom, since the most popular LMS platforms will likely face high pressure in the coming months ( Sutterlin, 2018; Martin and Tapp, 2019).

It is vital to communicate the plan to the college leadership early, so that they can provide feedback, approval, and alignment with the university’s strategic plan, and create a learning and work environment where faculty and students feel they have the power and initiative to participate effectively in the monitoring, evaluation, and improvement of the distance learning plan that is put in place ( Pollack and Pollack, 2015).

## Capacity building

The transition of the curriculum delivery from face-to-face instruction to distance/online learning requires a lot of time and work from both faculty and staff members. Capacity building programs should be run at the earliest possible stages of implementation. Faculty play various roles in online teaching, so training that builds these competencies among faculty should be provided ( Liu *et al.*, 2019). Development of this training should be based on the plan, a training needs assessment, and the planned mode of delivery. Four roles had been outlined for the e-teacher including; pedagogical, managerial, social, and technical ( Kwon *et al.*, 2019; Philipsen *et al.*, 2019; Zygouris-Coe, 2019). Capacity building needs to be accompanied by a post-implementation follow-up in order to address any possible gaps.

A more elaborate plan for capacity building needs to be designed and implemented after the emergency situation concludes, using the results of evaluation.

## Agree on curriculum content

The plan should accommodate the fact that whatever is implemented is part of an emergency plan. As such, course committees and course directors should decide on what to implement and any changes to the content - whether, through addition, omission, or postponement - and any new delivery methods should be adopted ( Clark, 2003; Patil and Chan Ho Yan, 2003).

Communication of the curriculum content should be done using a central body, such as a curriculum committee. The role of the working group is to facilitate the smooth communication of the content to staff and students. The following should be communicated early to students: timetables and study plans, e-problem-based learning case scenarios, instructions for e-practical labs, simulations, guidelines for online discussion forums, guidelines on virtual learning, and assessments.

Detailed descriptions of each session, whether synchronous or asynchronous, should be included on the timetables along with any reading materials and associated links that must be provided in advance to students.

Contact numbers and email addresses for faculty should also be communicated clearly to the students. Online office hours should be communicated so that students can seek out feedback from faculty members.

## Curriculum delivery

There are three main modes for implementing online learning: the synchronous, asynchronous, and blended modes. The third is beyond the scope of this article, but the other two will be addressed below.

The synchronous mode allows learners to engage in discussion with instructors and classmates using the LMS or LMS-equivalent application at the same time; the asynchronous mode allows learners to carry out discussions over the internet at different times. The latter does so by using tools such as discussion forums or email exchanges ( Ellaway and Masters, 2008). The synchronous option has the advantage of allowing for instant interaction and feedback, while the asynchronous mode allows for more control of pace and timing.

In the case of a sudden and forced curricular transition, in order to overcome the challenges of online teaching and related technical difficulties, the authors recommend beginning with the asynchronous mode. Simultaneously, training faculty in best practices for synchronous teaching could take place.

There are several tools available for the synchronous teaching of e-lectures e-problem-based learning, e-labs, and virtual patient ( Ellaway and Masters, 2008).

When teaching asynchronously, discussion forums and chatrooms can be used to enhance student engagement and interaction. Numerous strategies to increase participation have been proposed, including minimum numbers of posts, awarding marks for particular posts, or carefully constructing questions that are engaging. Awarding marks for particular posts is likely to increase the number of overall posts. However, these can be in the form of mini-assignments rather than requests for students’ spontaneous thoughts ( Lee and Ferwerda, 2017). Video games and the gamification of learning could also be used to engage and motivate students. There is a growing belief that the success of complex video games demonstrates that such games can teach higher-order thinking skills, such as strategic thinking, interpretative analysis, problem-solving, plan formulation and execution, and promote adaptation to rapid change ( Singhal, Hough and Cripps, 2019). The use of virtual patients is a key example of game-informed learning in medical education ( Lloyd *et al.*, 2017). Typically, virtual patients take the form of an open-ended clinical narrative or a structured patient encounter, the latter being more common. In either scenario, students may have to search for and/or interpret data, make appropriate clinical decisions, or solve problems such as making a diagnosis or formulating a treatment regimen.

There are several tools and LMS options that could be used for both synchronous and asynchronous online learning ( Ellaway and Masters, 2008), which are listed in table 1.

There are also simple, user-friendly social media tools such as WhatsApp, Telegram, or YouTube, all of which have demonstrated effectiveness as educational tools in medical education ( Jalali *et al.*, 2015; Raiman, Antbring and Mahmood, 2017; Sutherland and Jalali, 2017).

**Table 1.** Common tools to be used for online/distant learning (some of them are free)( Molly McLaughlin; Daniel Brame, 2020; Sutterlin, 2018; Baig, Gazzaz and Farooq, 2020)

Purpose	Tool	Link
**Discussion forum**	Blackboard	https://www.blackboard.com/
	Moodle	https://moodle.org
	Slack	https://slack.com/
	Schoology	https://www.schoology.com/
	Edmodo	https://www.edmodo.com/
	Flock	https://flock.com
**Online lecturing**	Zoom	https://zoom.us/
	Blackboard Collaborate Ultra	https://help.blackboard.com/Collaborate/Ultra
	Skype	https://www.skype.com/
	Google suite	https://gsuite.google.com/products/meet/
	Gotomeeting	https://www.gotomeeting.com/
	Go webex	https://www.webex.com/
	Bluejeans	https://www.bluejeans.com/
	Loom	https://www.loom.com/
	Teamviewer	https://www.teamviewer.com//
	Join.me	https://www.join.me/

## Dealing with students’ stress

In face-to-face learning, faculty can observe how their students work and learn and how they interact in the classroom. In online learning, however, it is more difficult for faculty to monitor individual students’ behavior and responses.

Several studies have revealed that online learning is more stressful for students than face-to-face learning ( Gillett-Swan, 2017). The design of courses can work to alleviate this stress.

In transitioning to online learning, teaching materials must be adapted for online delivery. Minimizing the number of synchronous sessions per day by providing recorded video content has been shown to be beneficial during a sudden shift to online teaching. Providing time for self-directed learning, as long as this has clear instructions, also minimizes student stress. Continuing academic mentoring and having devoted office hours for students is also important.

## Ensure student engagement and motivation

Because students in e-learning environments are more independent than face-to-face learners and content and activities are created and determined by faculty, instructors must do more to engage and motivate online learners.

Clear instructions, as well as pre- and post-exercises, are required to engage students with the teaching materials. Special care should be taken with planning and recording video content. The following measures are recommended when using video in medical education: orienting students to the video content; using interactive elements to promote student participation; aligning videos with learning objectives and course outcomes; integrating PowerPoint slides; including the lecturer’s image, on-screen captions, and a transcript; avoiding cognitive overload; and limiting video length ( Dong and Goh, 2015). Other activities, for instance multiple-choice questions and mini quizzes, have also been shown to be effective in engaging students taking part in online learning ( Irizarry, 2002).

## Consider student assessment

Assessing curriculum delivery using online/distant learning approaches should focus on the assessment of “formative” learning rather than “summative” learning. Thankfully, there are already numerous online tools that assist in this process, with the most common being exist Kahoot, Socrative, Quizlet Live, and Nearpod ( Baig, Gazzaz and Farooq, 2020). Interestingly, simple tools such as Google quizzes have proven to be just as effective in giving frequent feedback to students participating in online learning ( Anderson, 2019).

For summative assessment for the assessment of the knowledge domain, there are many options, including modified essay questions, assignments, and open book exams, using a variety of appropriate tools. Some LMS platforms provide portfolio tools, such as Taskstream ( https://www.watermarkinsights.com/), that allow learners to build online repositories of their work, experiences, and reflections over time, as well as to link to external images, documents, and media such as podcasts.

Assessment of the psychomotor domain is one of the most challenging in cases of online/distance learning. However, assessment of clinical reasoning can be done using virtual Objective Structured Clinical Examination (OSCE) stations and game worlds such as SecondLife ( Swicegood and Haque, 2015). Virtual patients can also provide many different ways to assess student performance ( Padilha *et al.*, 2019). Finally, there are various e-assessment resources that allow teachers to create questions and tests to assess student learning, such as QuestionMark Perception ( http://www.questionmark.com) and Respondus ( http://www.respondus.com) ( Danson, Dawson and Baseley, 2001; Küppers *et al.*, 2017).

## Anticipate challenges and plan for how to overcome them

Several challenges that the authors have faced during the implementation of online teaching, in the period of COVID-19, include the pressures on the LMS, internet connectivity at students’ locations, and a lack of skills among some faculty members or instructors regarding the use of specific online teaching technologies. The working team can recommend the following measures to help overcome these challenges: rescheduling the timetable; avoiding the use of synchronized online sessions at the same time, recording the sessions with the addition of discussion forum to engage the learners.

## Monitoring and evaluation of curriculum implementation and continuous improvement

Several tools can be used to monitor and evaluate the implementation of online teaching approaches. We recommend seeking student feedback following each session and obtaining daily feedback from faculty members in order to fully explore areas for improvement. This can be achieved with the use of a simple online questionnaire sent to all faculty that covers the following topics: the number of planned sessions, the number of implemented resources per session; the type of e-learning model/s used; comments on student attendance and interaction; the challenges faced during implementation; and suggestions on how to avoid these challenges. The results of these evaluations can be used to design an improved contingency plan for future emergency situations.

## Conclusion

The sudden transition from on-campus learning to distance learning approaches is a challenge for both faculty and students and has required a great deal of preparation over a short period of time.

A systematic approach with the involvement of the whole stakeholders is required for this change.

The proposed process will hopefully assist the medical schools in response to the current pandemic (COVID-19) and when facing similar situations.

## Take Home Messages

For the successful implementation of the transition to online/distant learning approach:

• Medical colleges have to follow a systematic approach.
• The Process including:
  • Establishing a sense of urgency,
  • Establishing working teams,
  • Conducting needs assessments,
  • Developing implementation plans,
  • Communicating the curriculum content,
  • Capacity building,
  • Managing students’ stress,
  • Finding tools to be used,
  • Managing student engagement and motivation,
  • Consider Student assessment,
  • Anticipating challenges and planning for how to overcome them, and
  • Monitoring and evaluation of curriculum implementation and continuous improvement.

## Notes On Contributors

**Mohamed Hassan Taha** s a visiting Assitant Professor of Medical Education at the College of Medicine and Medical Education Centre, University of Sharjah, UAE. He is the head of Faculty Development Committee. ORCiD: https://orcid.org/0000-0003-0808-5590.

**Mohamed Elhassan Abdalla** is an Assitant Professor of Medical Education at the College of Medicine and Medical Education Centre, University of Sharjah, UAE. He is the director of Medical Education Centre, chair of the curriculum committee. ORCiD: https://orcid.org/0000-0002-9241-1370.

**Majed Mohamed Saleh Wadi** is a senior lecturer of Medical Education, College of Medicine, University of Qassim, KSA. He is the Head of Medical Education Department. Head of the Assessment Committee. ORCiD: https://orcid.org/0000-0002-8117-770X.

**Husameldin Khalafalla** is an Assitant Professor of Community Medicine and Medical Education at the College of Medicine, University of Jizan, KSA. ORCiD: https://orcid.org/0000-0002-7643-6180.

## Declarations

The author has declared that there are no conflicts of interest.

## Ethics Statement

This manuscript is reflection on experience and practical guidelines.

## External Funding

This article has not had any External Funding

## References

[ref1] Ahmed H. Allaf M. and Elghazaly H. (2020) COVID-19 and medical education. The Lancet Infectious Diseases. Elsevier Ltd. 2019(20), p.30226. 10.1016/S1473-3099(20)30226-7 PMC7270510 32213335

[ref2] Anderson J. (2019) Frequent Feedback through Google Forms. PRIMUS. Taylor & Francis. 29(2), pp.124–137. 10.1080/10511970.2017.1411408

[ref3] Baig M. Gazzaz Z. J. and Farooq M. (2020) Blended Learning: The impact of blackboard formative assessment on the final marks and students’ perception of its effectiveness. Pakistan Journal of Medical Sciences. 36(3). 10.12669/pjms.36.3.1925 PMC7150402 32292428

[ref4] Clark J. (2003) Fear of SARS thwarts medical education in Toronto. BMJ (Clinical research ed.). 10.1136/bmj.326.7393.784/c PMC1169342 12689971

[ref5] COVID-19 Coronavirus Pandemic (2020). Available at: https://www.worldometers.info/coronavirus/( Accessed: 29 March 2020).

[ref6] Danson M. Dawson B. and Baseley T. (2001) Large Scale Implementation of Question Mark Perception (V2. 5). Experiences at Loughborough University. Loughborough University. Availableat: https://repository.lboro.ac.uk/articles/Large_Scale_Implementation_of_Question_Mark_Perception_V2_5_Experiences_at_Loughborough_University/9488513( Accessed: 29 March 2020).

[ref8] Dong C. and Goh P. S. (2015) Twelve tips for the effective use of videos in medical education. Medical teacher. Taylor & Francis. 37(2), pp.140–145. 10.3109/0142159X.2014.943709 25110154

[ref9] Ellaway R. and Masters K. (2008) AMEE Guide 32: E-Learning in medical education Part 1: Learning, teaching and assessment. Medical Teacher. 30(5), pp.455–473. 10.1080/01421590802108331 18576185

[ref10] Gillett-Swan J. (2017) The challenges of online learning: Supporting and engaging the isolated learner. Journal of Learning Design. Queensland University of Technology. 10(1), pp.20–30. Available at: https://files.eric.ed.gov/fulltext/EJ1127718.pdf( Accessed: 29 March 2020).

[ref11] Hafiz H. (2020) Regulating in Pandemic: Evaluating Economic and Financial Policy Responses to the Coronavirus Crisis. Boston College Law School Legal Studies Research Paper.(527). 10.2139/ssrn.3555980

[ref12] Irizarry R. (2002) Self-efficacy & motivation effects on online psychology student retention. Usdla Journal. 16(12). Available at: https://www.learntechlib.org/p/95843/( Accessed: 29 March 2020).

[ref13] Jalali A. (2015) Social media and medical education: exploring the potential of Twitter as a learning tool. International Review of Psychiatry. Taylor & Francis,27(2), pp.140–146. 10.3109/09540261.2015.1015502 25768325

[ref14] Küppers B. (2017) Beyond lockdown: towards reliable e-assessment. Bildungsräume 2017. Gesellschaft für Informatik, Bonn. Available at: https://dl.gi.de/bitstream/handle/20.500.12116/4841/B19%20Beyond%20Lockdown-%20Towards%20Reliable%20e-Assessment.pdf?sequence=1&isAllowed=y( Accessed: 29 March 2020).

[ref15] Kwon K. (2019) Effects of different types of instructor comments in online discussions. Distance Education. Taylor & Francis. 40(2), pp.226–242. 10.1080/01587919.2019.1602469

[ref16] Lee M. J. and Ferwerda B. (2017) Personalizing online educational tools.in Proceedings of the 2017 ACM Workshop on Theory-Informed User Modeling for Tailoring and Personalizing Interfaces.pp.27–30. 10.1145/3039677.3039680

[ref17] Liu X. (2019) Exploring Four Dimensions of Online Instructor Roles: a Program Level Case Study. Online Learning. 9(4), pp.29–48. 10.24059/olj.v9i4.1777

[ref18] Lloyd A. (2017) How to implement live video recording in the clinical environment: A practical guide for clinical services. International journal of clinical practice. Wiley Online Library,71(6), p. e12951. 10.1111/ijcp.12951 28524616

[ref19] Martin L. and Tapp D. (2019) Teaching with Teams: An introduction to teaching an undergraduate law module using Microsoft Teams. Innovative Practice in Higher Education. 3(3). Available at: http://journals.staffs.ac.uk/index.php/ipihe/article/view/188/284( Accessed: 29 March 2020).

[ref20] Molly McLaughlin; Daniel Brame (2020) The Best Video Conferencing Software for 2020. Available at: https://www.pcmag.com/picks/the-best-video-conferencing-software( Accessed: 29 March 2020).

[ref21] Padilha J. M. (2019) Clinical virtual simulation in nursing education: randomized controlled trial. Journal of medical Internet research. JMIR Publications Inc., Toronto, Canada,21(3), p. e11529. 10.2196/11529 30882355 PMC6447149

[ref22] Patil N. G. and Chan Ho Yan Y. (2003) SARS and its effect on medical education in Hong Kong. Medical Education. 10.1046/j.1365-2923.2003.01723.x PMC7168501 14984121

[ref23] Philipsen B. (2019) Improving teacher professional development for online and blended learning: A systematic meta-aggregative review. Educational Technology Research and Development. Springer. 67(5), pp.1145–1174. 10.1007/s11423-019-09645-8

[ref24] Pollack J. and Pollack R. (2015) Using Kotter’s eight stage process to manage an organisational change program: Presentation and practice. Systemic Practice and Action Research. Springer. 28(1), pp.51–66. 10.1007/s11213-014-9317-0

[ref25] Raiman L. Antbring R. and Mahmood A. (2017) WhatsApp messenger as a tool to supplement medical education for medical students on clinical attachment. BMC medical education. BioMed Central. 17(1), p.7. 10.1186/s12909-017-0855-x PMC5219809 28061777

[ref26] Singhal S. Hough J. and Cripps D. (2019) Twelve tips for incorporating gamification into medical education. MedEdPublish. 8. 10.15694/mep.2019.000216.1 PMC10712530 38089323

[ref27] Sun P. (2020) Understanding of COVID-19 based on current evidence. Journal of Medical Virology. 10.1002/jmv.25722 PMC7228250 32096567

[ref28] Sutherland S. and Jalali A. (2017) Social media as an open-learning resource in medical education: current perspectives. Advances in medical education and practice. Dove Press,8, p.369. 10.2147/AMEP.S112594 28652840 PMC5476438

[ref29] Sutterlin J. (2018) Learning is Social with Zoom Video Conferencing in your Classroom. eLearn. ACM. 2018(12), p.5.

[ref30] Swicegood J. and Haque S. (2015) Lessons from recruiting Second Life users with chronic medical conditions. Applications for health communications. Journal For Virtual Worlds Research. 8(1). 10.4101/jvwr.v8i1.7097

[ref31] UNESCO (2020) COVID-19 Educational Disruption and Response. Available at: https://en.unesco.org/themes/education-emergencies/coronavirus-school-closures( Accessed: 30 March 2020).

