# Supplementary material for: Curriculum delivery in Medical Education during an emergency: A guide based on the responses to the COVID-19 pandemic
Source: MedEdPublish (2016). 2020 Apr 16;9:69. [Version 1] doi: 10.15694/mep.2020.000069.1 (PMC10697579; doi:10.15694/mep.2020.000069.1)
Supplement: Peer review history

# Reviewer report: Reviewer response for version 1

**DOI:** 10.21956/mep.20033.r30614

**Published:** 2020-05-15

**Authors:** Elisabeth Schlegel

**Affiliations:** 1 Donald and Barbara Zucker School of Medicine at Hofstra/Northwell

This review has been migrated. The reviewer awarded 4 stars out of 5 I am thanking the authors for providing this “atlas” of change. This article outlines many important parameters and strategies necessary to manage the unexpected and challenging adaptations. Importantly, a leading team has to be established first in order to identify the resources needed (“sense of urgency”). Top leadership has to be on-board in order to guide executing the decisions downstream. Overall, many of the difficulties we face now can be compared to medical education in low-resource settings in several global locations. Here, home-grown developments and networking overcome many of the barriers. Thus, I want to encourage to inspire networking and creative thinking as much as possible under the circumstances. I wholeheartedly agree, transparency is key, and representatives from all stakeholder levels need to be included to master the challenges. This is a very comprehensive and timely article and we assigned it as pre-reading to our MS3 medical-student-as-teacher elective students prior to a session on Deliberate vs. Emergency Online Education.

Reviewer Expertise:

NA

No decision status is available

# Reviewer report: Reviewer response for version 1

**DOI:** 10.21956/mep.20033.r30616

**Published:** 2020-04-29

**Authors:** James Gray

**Affiliations:** 1 University of Sheffield

This review has been migrated. The reviewer awarded 3 stars out of 5 I think this presents a relatively useful toolkit for others to follow which is as much an approach to change management as a specific response to Covid19. Like another colleague has said the focus on the LMS perhaps slightly limits its application to those with less resource and broadening this to consider alternatives, or picking that up as a separate piece of work with colleagues in such environments might add some significant value. I would also have liked to see a little more in student stress about the communication process. My own experience has been that a significant contributor to student stress is uncertainty and so frank and open dialogue with them is an additional crucial component that then also lowers your own stress levels. Overall a useful read for anyone in a similar situation

Reviewer Expertise:

NA

No decision status is available

# Reviewer report: Reviewer response for version 1

**DOI:** 10.21956/mep.20033.r30622

**Published:** 2020-04-24

**Authors:** Samar Ahmed

**Affiliations:** 1 Ain Shams University Faculty of Medicine

This review has been migrated. The reviewer awarded 5 stars out of 5 Wonderful work and a guide that we will start introducing into our leadership in curriculum reform course

Reviewer Expertise:

NA

No decision status is available

# Reviewer report: Reviewer response for version 1

**DOI:** 10.21956/mep.20033.r30621

**Published:** 2020-04-24

**Authors:** Dujeepa D. Samarasekera

**Affiliations:** 1 National University of Singapore

This review has been migrated. The reviewer awarded 4 stars out of 5 I enjoyed reading this article. Very comprehensive and detailed. The COVID 19 pandemic has changed the way higher education centres engage students and other stakeholders. The article will assist many places to design their teaching-learning environment. Useful take-home messages.

Reviewer Expertise:

NA

No decision status is available

# Reviewer report: Reviewer response for version 1

**DOI:** 10.21956/mep.20033.r30612

**Published:** 2020-04-19

**Authors:** David Taylor

**Affiliations:** 1 Gulf Medical University, Ajman, UAE

This review has been migrated. The reviewer awarded 4 stars out of 5 This paper is valuable, since it gives some idea of the large number of IT resources which are available. Like other reviewers I noted the reliance on ensuring that one has a learning management system – I would add that institutions with a LMS probably need thestaff who can, with a degree of authority based on experience, be able to maintain and adjust such a system. I whole heartedly agree with the take home messages although the issues in delivering them would probably warrant a paper on each item. Well done though for such a concise and timely contribution.

Reviewer Expertise:

NA

No decision status is available

# Reviewer report: Reviewer response for version 1

**DOI:** 10.21956/mep.20033.r30620

**Published:** 2020-04-18

**Authors:** Sarmishtha Ghosh

**Affiliations:** 1 International Medical University

This review has been migrated. The reviewer awarded 4 stars out of 5 In the current situation, when there is a flooding of websites and tools for online teaching-learning for delivery of the curriculum, this article provides specific lists of tools that can be used by educators to deliver the curriculum to the students. The other challenge is how to maintain students' interest and keep them engaged throughout the course. The authors have addressed that issue also categorically which gives the readers an idea of how to implement. Of course, the authors have not shared their own experiences but then delivery is contextual. Hence educators can choose what works best for them and evaluate regularly to improve upon the existing ones. Pandemic has actually created an emergency remote learning situation rather than a planned online learning process. So this article can help people to design online delivery of curriculum, wherever possible, even after the Pandemic is gone, in addition to this quick transition of different formats. The list of "take-home messages" is of particular interest.

Reviewer Expertise:

NA

No decision status is available

# Reviewer report: Reviewer response for version 1

**DOI:** 10.21956/mep.20033.r30611

**Published:** 2020-04-18

**Authors:** Susanne Lindqvist

**Affiliations:** 1 University of East Anglia

This review has been migrated. The reviewer awarded 5 stars out of 5 Thank you for sharing this paper, which includes a number of tools that I will be keen to explore. Universities across the world will have had to rapidly change their approach to education in response to the COVID-19 crisis, and we all know that once this is over the teaching will look different in the future, with more online and blended learning opportunities. The paper serves as helpful guide of key elements that we need to consider in this transition, including the need for team working and making sure everyone has the necessary equipment to connect and indeed knows how to. Moving forward, this paper reminds us of the importance of really think about the impact on this crisis, not only in the in the short- but also the long-term and whether some learning that was previously face-to-face is actually better delivered online. Saying that, I still hope that the ‘going virtual’ is going ‘viral’ in a manner that allows us to find balance and truly understand how students learn in the most effective way and how we as teachers evolve alongside. The take home message was very useful to aid this process thank you.

Reviewer Expertise:

NA

No decision status is available

# Reviewer report: Reviewer response for version 1

**DOI:** 10.21956/mep.20033.r30619

**Published:** 2020-04-17

**Authors:** Judy McKimm

**Affiliations:** 1 Swansea University

This review has been migrated. The reviewer awarded 3 stars out of 5 I enjoyed this summary of tips for educators to consider during these challenging times. Like some of the other reviewers I felt that reliance on buying an LMS or other IT infrastructure isn’t relevant to or possible for those in low resources settings and also that it somewhat simplified what is a very complex set of issues which will probably extend into next academic year for those of us in the global north. However, these resources and quick publications are useful for educators to tap into whilst they are giggling difficult decisions for learners and faculty.

Reviewer Expertise:

NA

No decision status is available

# Reviewer report: Reviewer response for version 1

**DOI:** 10.21956/mep.20033.r30615

**Published:** 2020-04-17

**Authors:** P Ravi Shankar

**Affiliations:** 1 American International Medical University

This review has been migrated. The reviewer awarded 4 stars out of 5 I enjoyed reading this article on teaching-learning during the COVID-19 pandemic. The pandemic has had a severe impact on various aspects of life and teaching-learning has also been affected. The authors provide a step-by-step guide to curriculum delivery in medical education. They provide links to a number of resources which will be useful to readers. The suggestions provide a broad framework which can be further developed by the readers. The list of references is comprehensive. The article will be of interest to all medical educators during the present challenging time.

Reviewer Expertise:

NA

No decision status is available

# Reviewer report: Reviewer response for version 1

**DOI:** 10.21956/mep.20033.r30618

**Published:** 2020-04-17

**Authors:** Johnny Lyon Maris

**Affiliations:** 1 Southampton GP Education Unit

This review has been migrated. The reviewer awarded 4 stars out of 5 Thankyou for submitting this article which is a very useful compendium of the on-line resources available. I would however challenge Figure 1 where you suggest that if the higher education institution does not have a Learning Management System, then it should purchase one. This is a very costly intervention, and for some Universities, not affordable on the scale you suggest. It also relies on fast internet which can also be problematic if the students are in rural and remote areas or attachments. This is not a developing world problem, here in the UK we have problems across the country with internet availability and speed.

Reviewer Expertise:

NA

No decision status is available

# Reviewer report: Reviewer response for version 1

**DOI:** 10.21956/mep.20033.r30613

**Published:** 2020-04-17

**Authors:** Kitiyot Yotsombut

**Affiliations:** 1 Chulalongkorn University

This review has been migrated. The reviewer awarded 5 stars out of 5 Although experiences in using such approaches as well as the mentioned tools have not been discussed in detail, this article has provided useful approaches regarding online distance medical learning in COVID-19 era.

Reviewer Expertise:

NA

No decision status is available

# Reviewer report: Reviewer response for version 1

**DOI:** 10.21956/mep.20033.r30617

**Published:** 2020-04-16

**Authors:** Trevor Gibbs

**Affiliations:** 1 AMEE

This review has been migrated. The reviewer awarded 4 stars out of 5 As expected, we are seeing an increasing number of papers about managing and coping during the Covid-19 pandemic and all of them provide a good insight into various approaches. This is one such paper seemingly based upon a 12 Tips approach. I enjoyed reading it and it brought out some very salient features. At points I did worry about the superficiality of each of the suggestions and probably left the reader waiting or wanting for more. It does provide however a very good list of references.

Reviewer Expertise:

NA

No decision status is available
